# Atlanta Medical College

**Published:** 1883-05

**Authors:** 


### ATLANTA MEDICAL COLLEGE.

At the recent Commencement of this institution, the annual address was delivered by Judge Logan E. Bleckley. It was a brilliant medley of prose and poetry.

The following is the poetical part of the address conveying valuable instruction to those most interested, by an inimitable play of words in the life story of a well known, and most ancient *practitioner*.

By those who are well acquainted with the overflowing geniality and sparkling wit of the man, it will be especially relished:

His subject was "Patience."

The question of questions is now to be met;
The practical question, how patients to get?
The patience of Job, since Job has retired,
May either be purchased or borrowed or hired;
The first thing to ponder—to ponder and probe,
Is, how to acquire the patience of Job.
When he was in practice his patience was much;
And every young doctor needs plenty of such—
The patience to labor, to learn, and to wait,
To suffer, endeavor, endure, and be great.
Job's practice was simple; his treatment was what?
Some ashes, and scraping with pieces of pot;
Then silence a week; then cursing a day;
Then railing at everything others could say.
How unscientific! and yet, as we read,
No practice than his did could better succeed.
The patient recovered—remained on the stage
Through four generations, and died of old age.
The fee was a fortune! He got, we are told,
Unlimited money and ear-rings of gold—
With camels, and asses, and oxen, and sheep,
By thousands—much more than his pasture would keep.
Ah! Job had the patience, the time and the place!
His riches, remember, were all for one case.
Whatever you covet, or merit or earn,
Depend upon patience to make the return.
Your science and learning, you latest M.D.'s,
Your medals and honors, diplomas, degrees—
Why, Job's were as nothing contrasted with these,
But they never will bring in such fabulous fees.
If doctors have patience, the patients will come;
Some can not have all, but they all may have some;
If patients have patience, 'tis safe to fortell
That twelve to the dozen are sure to get well.
Whoever says "patients" has "patience" to say;
By name and by nature, in both there is pay.
That like will cure like, is the hint that peeps through;
"Similia similibus curantur," is true.

---

WALKING, a few days ago, on Whitehall street, we overtook our friend Dr. C., of this city, just in the rear of a group of high school girls returning from school.

"Do you observe that?" said the Doctor, touching our arm.

"Observe what, Doctor?"

"Why, the curvature of that girl's spine there. Do you not see at a glance that it is now almost in the shape of the letter S?"

Sure enough there was the zigzag deformity plainly to be seen by anyone approaching her from the rear.

The subject of this observation appeared to be some fifteen years of age, and with the exception of a certain listlessness in her step seemed healthy enough. Doubtless the young lady is wholly unconscious of the serious condition of her spinal column, and what is equally probable, and more to be lamented, her parents and teachers are as ignorant of it as herself.

"But, Doctor, this I suppose is only an exceptional case among the otherwise robust girls of Atlanta."

"No sir, you are quite mistaken. This spine curvature is getting to be a frequent abnormality of the school girls of our day. I do not say that it occurs oftener in Atlanta than in other cities, either North or South, but I do say that I have frequently observed it here, and regard it as one of the most serious evils resulting from the pushing, cramming, hot-house system of public school education now so much in vogue everywhere."

We do not object—we fell a musing after leaving the Doctor—to education both public and private; but is there not, after all, a higher culture for our children, by which their precious endowment of health is preserved, with a lower development of the merely intellectual?

Will Southern parents, teachers and educational boards practice what they know to be true; that what is now popularly called education, if acquired at the expense of physical health, is obtained at a price fearfully beyond its real worth.

They should take warning from the fact that while the women of New England are remarkable for their proficiency in natural science and metaphysics, they have become so lank and lean in body as to elicit from the impudent young men there the oft-repeated social toast, "Beauty and bones."

We gladly welcome all that is good from New England, but may heaven defend us from an importation of bones and metaphysics, or any system that will set up a manufactory of them in the South.

